# A case of gastric metastasis originating from right-sided colon cancer 4 years after colectomy

**DOI:** 10.2144/fsoa-2022-0016

**Published:** 2022-11-01

**Authors:** Amal Khsiba, Manel Moalla, Moufida Mahmoudi, Asma Ben Mohamed, Yaakoubi Manel, Salwa Nechi, Houda Belfekih, Selim Zribi, Mouna Medhioub, Lamine Hamzaoui, Mohamed Moussadek Azzouz

**Affiliations:** 1 Department of Gastroenterology, Mohamed Taher Maamouri Hospital, Nabeul, Tunisia; 2 Department of Cytology, Mohamed Taher Maamouri Hospital, Nabeul, Tunisia; 3 Department of Oncology, Mohamed Taher Maamouri Hospital, Nabeul, Tunisia; 4 Department of Surgery, Mohamed Taher Maamouri Hospital, Nabeul, Tunisia

**Keywords:** colon adenocarcinoma, colorectal cancer, gastric metastases

## Abstract

The stomach is rarely a metastatic site of other primary cancers. Gastric metastasis from colonic cancer is exceptional. We hereby report a case of a 54-year-old male patient who underwent a right hemicolectomy for right-sided colon cancer. The pathology exam revealed well differentiated adenocarcinoma, it was classified stage IIb. Regular controls performed including colonoscopy were normal. Four years after colectomy, the patient was admitted for hematemesis with epigastric pain with detoriation of general condition . Gastroscopy revealed a large ulceroproliferative mass in the antropyloric region. Histology showed that this tumor was an adenocarcinoma similar to the primary right colon cancer, which led to the diagnosis of metastatic gastric cancer originating from colon cancer.

## Plain language summary

Gastric metastasis from colonic cancer is exceptional. We hereby report a case of a 54-year-old male patient who underwent a right hemicolectomy for right colon cancer. The pathology exam revealed well differentiated adenocarcinoma stage IIb. Four years after the right hemicolectomy, the patient was admitted for hematemesis and epigastric pain with worsening of general state. Gastroscopy revealed a large ulceroproliferative mass in the antropyloric region that corresponded histologically to gastric metastasis of colon cancer.

Metastases are the major cause of death in patients with colorectal cancers (CRC). Approximately, 20% of CRC present with metastases at diagnosis. The most common sites of metastases are liver, lung and peritoneum. Gastric metastasis is exceptional with few cases reported in medical literature. Signet-ring cell carcinoma seems to be more likely responsible of metastases to uncommon sites in patients with CRC [1]. However, the pathogenesis of gastric metastasis from CRC remains unclear. We report a case of a right-sided colon cancer with metachronous metastasis to the stomach.

## Patient presentation

**Figure 1.**
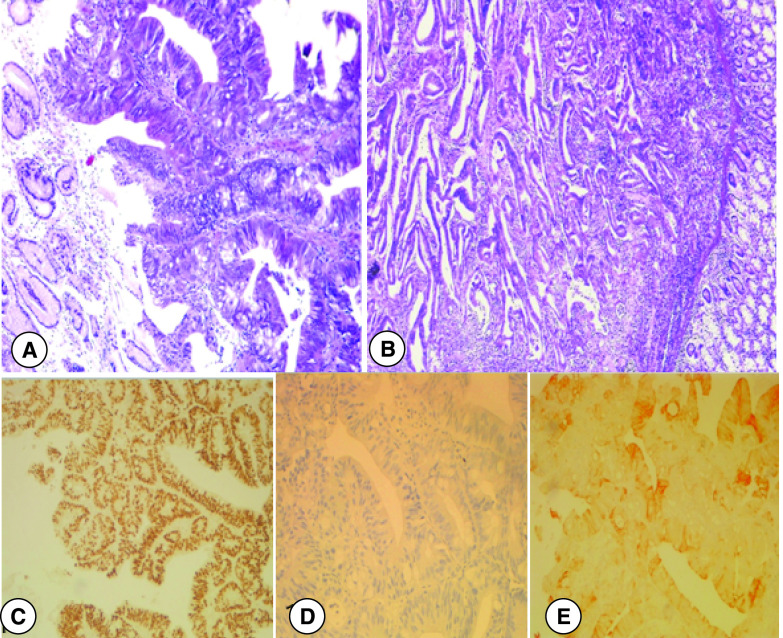
Pathological examination of primitive tumor and metastasis.

**Figure 2.**
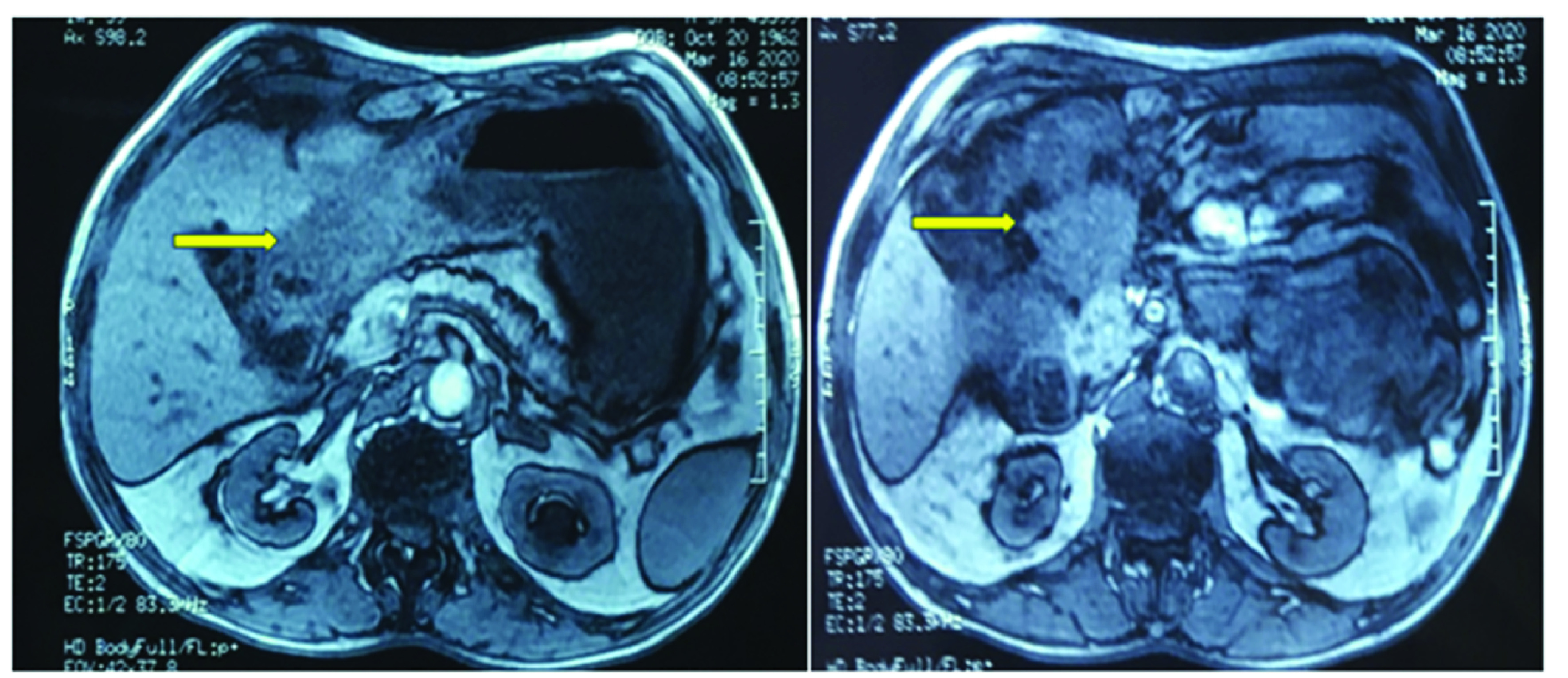
MRI showing a mass at the expense of the anastomosis invading the antropyloric region and coming into contact with the head of pancreas and the left liver lobe.

A 54-year-old male patient was diagnosed with a stenotic right-sided colon cancer. Tumor was a well differentiated adenocarcinoma classified stage IIb. Intraoperative findings were a right-sided colonic mass without any signs of local or distant spread. He underwent a right hemicolectomy after which he had regular controls including colonoscopies. About 4 years after, he presented with upper digestive tract bleeding with a poor general state. Biological tests showed severe anemia (hemoglobin at 7.3 g/dl) and renal dysfunction (creatinine level at 329 µmol/l). Upper endoscopy revealed a largeulcero proliferative mass in the antropyloric region with normal overlying mucosa. Histological findings of gastric tumor (Figure 1A) were perfectly stackable with the samples of the colon cancer previously examined (Figure 1B). Immunohistochemical staining was highly positive for CDX2 (Figure 1C), negative for CK7- (Figure 1D) and positive for CK20+ pattern (Figure 1E) which consolidated the diagnosis of gastric metastasis originating from colon cancer. Colonoscopy revealed a diminutive anastomotic polyp. Abdominal MRI (Figure 2) was performed since the patient had severe renal failure. It showed a 7 cm invasive tumor of anastomosic colic region invading the stomach, duodenum and coming in contact with the head of pancreas and the left liver lobe evocative of a recurrence of the colon cancer.

He was planned for a gastroentero anasomosis. Intraoperatively, multiple abdominal adhesions and a voluminous mass in the epigastric region and in the right hypochondrium invading the terminal ileum and the colic anastomosis were found. Ileo sigmoidal anastomosis was performed. Then the patient underwent three cycles of chemotherapy based on FOLFOX. Follow-up period was 13 months after recurrence diagnosis. The cause of death was a severe anemia refractory to blood transfusions.

## Discussion

Gastric metastasis is an uncommon situation with an incidence ranging from 0.2 to 0.7% in the literature [2,3]. The most common primary tumors that metastasize to the stomach are lung cancer, breast cancer and melanoma [4]. A study reported the case of 20 patients with gastric metastasis from different primary origins, the mean interval of diagnosis was of 16 months (0–56 months) from the time of diagnosis of the primary neoplasm to the diagnosis of the gastric metastases [5]. In the majority of their patients, the diagnosis was established in less than a year. Metastasis from CRC to the stomach is exceptional [6]. The pathogenesis of gastric metastasis from CRC remains unclear, Some authors believe that patients develop widespread lymph nodal metastases [7]. To the better of our knowledge, only 18 cases (including ours) have been reported in the literature so far. Majority of cases (15) were from Japan, with one case from Macedonia and one case from South Korea. Table 1 summarizes the mean characteristics of these cases. The mean age of metastasis diagnosis was 63.9 years, ranging from 35 to 82 years old. They were divided into ten men and eight women. The primary cancer was located in the right, transverse, left, sigmoid colon and in the rectum in 4, 6, 1, 4 and 3 patients, respectively. In all cases, the gastric tumor had a submucosal development. The gastric metastasis was unique in 15 cases and multiple in three cases. It was located in the stomach as follow: 5 in the upper third, 4 in the middle third, 7 in the lower third and 2 in the whole stomach. The metastasis was synchronous in eight cases and metachronous in ten cases. The mean interval from the time of diagnosis of the primary cancer to the diagnosis of the gastric metastasis was about 13.1 months (0–48 months). The originality of our case consists in the delay of 4 years between curative surgery for stage II colon cancer and the gastric metastasis revealed by gastrointestinal bleeding. Thirteen patients including our patient had another metastasis location besides the gastric one. The most common locations were the lymph nodes (n = 6), the lungs (n = 4) and the intestine (n = 3). The diagnosis is based on the histological exam. Samples from the colonic and the gastric cancer show usually the same features [5]. When immunohistochemical staining is positive for CDX2 and CK20, and negative for CK7, it supports the diagnosis of gastric metastasis from CRC with a higher a specificity for the CK7-/CK20+ pattern [ 4,20,21]. Our patient had a CK7-/CK20+ pattern and a positive staining for CDX2. Concerning therapy, it is well established that in case of metastases of CRC to the lung or liver, curative surgery should be performed when resectable. However, when metastases concerns unusual sites such as stomach, approach is not consensual [5]. The prognosis is generally poor since gastric involvement occurs in an advanced stage and is usually accompanied with other metastatic locations [6]. In fact, published studies showed that most of the patients had a limited survival of approximately few months. Only two studies reported a long survival: the longest one being about 7 years for a Japanese patient [5,9].

**Table 1.** Reported cases of gastric metastasis originating from colorectal cancer.

Study	Year	Age	Sex	Colon	Stomach	Number	Delay	Time	Other metastasis	Ref.
Nobusawa *et al.*	1998	68	F	T	M	Unique	S	0	PUL, LYM	[7]
Shida *et al.*	2003	71	F	T	L	Unique	M	40	0	[8]
Yoshimi *et al.*	2007	74	F	S	U	Unique	M	24	HEP, PUL	[9]
Sakabe *et al.*	2007	53	M	Rec	UML	Multiple	M	14	HEP,PUL,INT,BN	[10]
Onoe *et al.*	2008	35	F	R	L	Unique	S	0	0	[11]
Toyota *et al.*	2008	65	M	R	UML	Multiple	S	0	ESO, BN, REC	[12]
Shirakawa *et al.*	2009	73	M	S	U	Unique	M	39	ABD, LYM	[13]
Nozaki *et al.*	2009	76	M	Rec	L	Multiple	M	7	LYM	[14]
Sano *et al.*	2010	67	M	S	U	Unique	M	35	LYM	[15]
Iino *et al.*	2014	48	M	Rec	L	Unique	S	0	0	[16]
Nushijima *et al.*	2014	52	F	T	L	Unique	M	15	PER, LYM	[6]
Mori *et al.*	2015	57	F	Le	M	Unique	M	5	INT	[17]
Yoshino *et al.*	2017	76	M	S	M	Unique	S	0	0	[18]
Terashima *et al.*	2019	61	F	T	M	Unique	S	0	0	[5]
Our case	2020	54	M	R	L	Unique	M	48	INT	
Lee *et al.*	2020	82	M	R	U	Unique	M	9	JEJUNAL	[19]
Iwai *et al.*	2020	76	F	T	U	Unique	S	0	PUL, SKIN	[1]
Gligorievski *et al.*	2021	63	M	T	L	Unique	S	0	Omentum, LYM	[20]

ABD: Abdominalwall; BN: Bone; F: Female; HEP: Hepatic; INT: Intestine; L: Lowerthird; Le: Left; LYM: Lymphnodes; M: Male; M: Metachronous; M: Middle third; PER: Peritoneum; PUL: Pulmonary; R: Right; Rec: Rectum; S: Sigmoid; S: Synchronous; T: Time in months; T: Transverse; U: Upperthird.

## Conclusion

Few cases of gastric metastasis of colon cancer are reported. Our case report exposes a rare metastatic location 4 years after surgery of colonic cancer, the lesson that should be learned is to realize an upper endoscopy in the follow-up of patients with colonic cancer especially if associated to symptoms such as epigastric pain, vomiting or other gastric symptoms.

**Executive summary**

- The stomach is a rare site of metastases.
- The most common primary tumors that metastasize to the stomach are lung cancer, breast cancer and melanoma.
- The mean interval of diagnosis is 16 months.
- Positive staining for CDX2 and CK20 and negative staining for CK7, supports the diagnosis of gastric metastasis from colorectal cancer with a higher a specificity for the CK7-/CK20+ pattern.
- The prognosis is generally poor.
